# ACPAs Are Much More Than Diagnostic Autoantibodies

**DOI:** 10.5041/RMMJ.10255

**Published:** 2016-10-31

**Authors:** Abdulla Watad, Howard Amital

**Affiliations:** Department of Medicine “B”, Sheba Medical Center, Tel-Hashomer, Israel; Zabludowicz Center for Autoimmune Diseases, Sheba Medical Center, Tel-Hashomer Israel; and Sackler Faculty of Medicine, Tel-Aviv University, Tel-Aviv, Israel

**Keywords:** ACPA, anti-CCP, citrullination, rheumatoid arthritis

## Abstract

Anti-citrullinated protein autoantibodies (ACPAs) are the major autoantibodies in rheumatoid arthritis (RA). Anti-citrullinated protein autoantibodies are directed against different citrullinated antigens, including filaggrin, fibrinogen, vimentin, and collagen. Presence of ACPA is associated with joint damage and extra-articular manifestations, suggesting that ACPAs are most likely pathogenic autoantibodies in RA. *In vitro*, ACPAs induce macrophage tumor necrosis factor alpha (TNF-α) production, osteoclastogenesis, and complement activation. These autoantibodies also induce the formation of neutrophil extracellular traps (NETs). Additionally, ACPAs induce pathogenic cytokines expression and oxidative stress in immune cells derived from RA patients. The aim of this review is to show the pathogenic roles of these autoantibodies in RA.

## INTRODUCTION

Rheumatoid arthritis (RA) is an autoimmune disease that affects nearly 1% of the worldwide adult population, characterized by progressive synovial inflammation resulting in irreversible joint destruction. Many of the RA-specific autoantibodies are generated against citrullinated antigens and termed anti-citrullinated protein autoantibodies (ACPAs).^1^–^3^ Anti-citrullinated protein autoantibodies can be detected in almost 80% of RA patients^4^ and can be detected years before the onset of symptoms; moreover, their presence was shown to correlate with joint inflammation in RA.^5^,^6^ Anti-citrullinated protein autoantibodies are used as diagnostic markers of RA.^7^,^8^ In ELISA tests ACPA reaches high sensitivity and remarkable specificity levels for RA over other autoimmune conditions.^9^ The assay has since been optimized, and it is included among the revised 2010 classification criteria for RA.^10^ Recently a new autoantibody system was reported, and anti-carbamylated protein (anti-CarP) antibodies have been identified in RA patients.^11^ These autoantibodies have been associated with a worse disease progression independent of ACPAs.

The major targets for ACPAs are citrullinated peptides, i.e. peptides which have undergone post-translation conversion of arginine to citrulline-based residues. In RA, many proteins, such as filaggrin, fibrinogen, vimentin, and collagen type II, are subjected to citrullination.^12^ Currently, citrullinated peptides serve merely as a biomarker for diagnosis of the arthritic disease. These peptides contain a unique amino acid, citrulline, and alter the protein structure within the connective tissue, leading to tolerance breakdown and triggering the autoimmune response in RA.^13^

In humans, there is a close relationship between ACPA titers and inflammation as well as oxidative stress associated to RA.^14^ Several publications have shown that ACPAs are responsible for pathogenic mechanisms of RA. Anti-citrullinated protein autoantibodies were shown to induce inflammatory TNF-α production.^15^–^18^ Anti-citrullinated protein autoantibody levels were found to correlate with enhanced NETosis (neutrophils extracellular traps) in RA.^19^ Autoantibodies against mutated citrullinated vimentin (i.e. MCV-ACPAs) have been shown to enhance osteoclast precursor differentiation into mature bone-resorbing cells and bone loss.^20^ Although ACPAs have not been shown to induce arthritis by itself in animal models, the pathogenic effect of ACPAs was shown in experimental murine models of arthritis.^21^,^22^

Citrullinated peptides are used for diagnosis of RA by identifying the presence of ACPAs. The citrullinated peptides represent highly accurate antigenic ligands for the ACPA-ELISA test.

## STUDIES ON PATHOGENIC ROLE OF ACPAS IN RA

Several studies have reported the contribution of ACPAs in the pathogenesis of RA. A cohort of 238 patients with RA was followed longitudinally for 10 years with the collection of clinical data and serum samples; of them 125 patients with radiographs of the hands available at both baseline and after 10 years were included in this study. Radiographs were scored according to the van der Heijde modified Sharp score. Baseline sera were analyzed for C-reactive protein (CRP), erythrocyte sedimentation rate (ESR), ACPAs, IgA-RF and IgM-RF. Logistic regression analyses were used to identify predictors of radiographic progression and to examine the effect of ACPA level. Presence of ACPAs (odds ratio (OR) 4.0; 95% confidence interval (CI) 1.6 to 10.0) was the strongest independent predictor of radiographic progression. Female gender (OR 3.3; 95% CI 1.3 to 7.6), high ESR (OR 3.2; 95% CI 1.2 to 7.6), and a positive IgM-RF (OR 3.1; 95% CI 1.2 to 7.9) were also independent predictors. Compared with the ACPA-negative patients, patients with low to moderate levels of ACPAs (OR 2.6; 95% CI 0.9 to 7.2) and patients with high levels of ACPAs (OR 9.9; 95% CI 2.7 to 36.7) were more likely to develop radiographic progression.^6^

Another study investigated the value of ACPAs for predicting joint outcomes in patients with early RA; 191 patients were followed up prospectively for five years. Serum samples obtained from 145 patients at baseline before disease-modifying anti-rheumatic drug treatment were examined using three anti-citrullinated protein/peptide antibody assays: antiperinuclear factor (APF) by indirect immunofluorescence (IIF), antikeratin antibodies (AKA) by IIF, and ACPAs by enzyme-linked immunosorbent assay (ELISA). Radiographs of the hands and feet taken at baseline and after three and five years were evaluated using Sharp scores modified by van der Heijde. The likelihood of a total Sharp score increase after five years was significantly greater among patients with ACPAs (67%; OR 2.5; 95% CI 1.2 to 5.0) or APF (57%; OR 2.4; 95% CI 1.2 to 4.9), but not rheumatoid factor (OR 0.7; 95% CI 0.3 to 1.5). Mean values for radiographic damage, erosion, and joint narrowing scores at the three times were significantly higher in patients with ACPAs or APF than in those without. AKA did not significantly predict radiographic damage. In separate analyses of patients with and without RF, ACPAs or APF was better than RF for predicting total joint damage and joint damage progression after five years.^23^

A study by Kundu et al.^14^ aimed to establish whether measurement of the redox status in blood mirrors the oxidant status at sites of inflammation in patients with RA. The authors concomitantly examined their oxidant status by spectrophotometry and/or flow cytometry. The study reported that raised levels of superoxide in neutrophils of synovial infiltrate showed a positive correlation with NADPH oxidase activity in synovial fluid. Additionally, as reactive oxygen species generated in both peripheral blood and synovial infiltrate correlated positively with both DAS 28 and CRP/ACPAs levels, its measurement can serve as an indirect measure of the degree of inflammation in patients with RA. An *in vitro* human model was developed in which monocyte-derived macrophages were stimulated with ACPA-containing immune complexes (ICs) that were generated by capturing ACPAs from RA sera on immobilized citrullinated fibrinogen. Cellular activation was evaluated by TNF-α assay in culture supernatants. The ACPA-containing ICs induced a dose-dependent TNF-α secretion by macrophages from 14 of 20 healthy donors. The macrophage response was systematically higher than that of the paired monocyte precursors. The TNF-α secretion was not reduced by blockade of Fc gammaRI or Fc gammaRIII, but was strongly repressed when interaction of ICs with Fc gammaRII was prevented. The two citrullinated peptides significantly inhibited ACPA reactivity to citrullinated fibrinogen and, when tested together, almost completely abolished formation of macrophage-activating ICs, thereby diminishing the secreted TNF-α levels.^15^ Another *in vitro* study used ACPAs that were purified from pooled ACPA-positive RA sera by cyclic citrullinated peptide-conjugated affinity column. After culture of U937 cells with ACPAs, the TNF-α production and NF-kappaB DNA-binding activity of the cells were measured by ELISA. The cognate antigens of ACPAs on the U937 cell surface were probed by ACPAs, and the reactive bands were examined via proteomic analysis. Anti-citrullinated protein autoantibodies specifically enhanced TNF-α production and increased the DNA-binding activity of NF-kappa B in U937 cells. Proteomic analysis revealed that Grp78 protein (72 kd) was one of the cognate antigens of ACPAs. The truncated form of cell surface-expressed Grp78 (55 kd) on U937 cells contained citrulline capable of binding with ACPAs. After citrullination, glutathione S-transferase-tagged recombinant Grp78 (97.52 kd) became a 72-kd fragment and bound with ACPAs. The ACPAs also bound to human monocytes and lymphocytes and enhanced TNF-α production.^16^

A study by Khandpur et al.^19^ investigated whether aberrant NETosis occurs in RA, determined its triggers, and examined its deleterious inflammatory consequences. Enhanced NETosis was observed in circulating and RA synovial fluid neutrophils compared to neutrophils from healthy controls and from patients with osteoarthritis. Further, netting neutrophils infiltrated RA synovial tissue, rheumatoid nodules, and skin. NETosis correlated with ACPA presence and levels and with systemic inflammatory markers. Rheumatoid arthritis sera and immunoglobulin fractions from RA patients with high levels of ACPA and/or rheumatoid factor significantly enhanced NETosis, and the neutrophil extracellular traps (NETs) induced by these autoantibodies displayed distinct protein content. Indeed, during NETosis, neutrophils externalized the citrullinated autoantigens implicated in RA pathogenesis, and anti-citrullinated vimentin antibodies potently induced NET formation. Moreover, the inflammatory cytokines interleukin (IL)-17A and TNF-α induced NETosis in RA neutrophils. In turn, NETs significantly augmented inflammatory responses in RA and osteoarthritis synovial fibroblasts, including induction of IL-6, IL-8, chemokines, and adhesion molecules. These observations implicate accelerated NETosis in RA pathogenesis, through externalization of citrullinated autoantigens and immune stimulatory molecules that may promote aberrant adaptive and innate immune responses in the joint and in the periphery, and perpetuate pathogenic mechanisms in this disease.^19^ Recently we constructed a multi-antigenic peptide for immune tolerance specific for RA. The peptide is composed of a sequence of known citrullinated autoantigens (filaggrin, β-fibrinogen, collagen type II, and vimentin) in analogy to the mix of citrullinated peptides that are used currently in diagnostic sera tests. This multi-epitope citrullinated peptide we tailored was injected to adjuvant induced arthritis rats and was shown to attenuate arthritis manifestations. The amelioration in clinical signs of arthritis was related to up-regulation of a T regulatory cell subset and an elevated apoptosis rate of T cells associated with reduced Th17 population.^24^ A recent study by Seaman et al. found a correlation between anti-PAD3 antibodies and joint erosion score (JES).^25^ The same cohort showed no correlation between the measured ACPAs and JES.

In summary, ACPAs are the most specific autoantibody markers in patients with rheumatoid arthritis (RA) and seem to correlate with the disease activity. ACPA-positive RA patients suffer from an erosive and more aggressive disease compared to ACPA-negative patients; however, the role of ACPAs in RA pathogenesis is becoming more and more understood in the last years. Anti-citrullinated protein autoantibodies have diverse pathogenic effects including the induction of macrophage TNF-α production, osteoclastogenesis, and complement activation, and patients with high levels of ACPA are more likely to develop radiographic progression in the joints. The fact that ACPAs are a part of the pathogenesis of RA may make them a potential target of therapy of the disease.

## Abbreviations

ACPAs: anti-citrullinated protein autoantibodies
AKA: antikeratin antibodies
APF: antiperinuclear factor
CRP: C-reactive protein
ELISA: enzyme-linked immunosorbent assay
NETs: neutrophil extracellular traps
RA: rheumatoid arthritis
TNF-α: tumor necrosis factor alpha
